# Automated Rayleigh-Wave Nonlinear Acoustic Platform for Real-Time Fatigue Monitoring in Metallic Materials

**DOI:** 10.3390/s26103190

**Published:** 2026-05-18

**Authors:** Theodoti Z. Kordatou, Spyridoula G. Farmaki, Dimitrios A. Exarchos, Theodore E. Matikas

**Affiliations:** Mechanics, Smart Sensors & Nondestructive Evaluation (MSS-NDE) Laboratory, Department of Materials Science and Engineering, University of Ioannina, 45110 Ioannina, Greece; s.farmaki@uoi.gr (S.G.F.); matikas@otenet.gr (T.E.M.)

**Keywords:** nonlinear ultrasonics, Rayleigh surface waves, LabVIEW automation, fatigue monitoring, Laser Doppler vibrometry, real-time signal processing, structural health monitoring

## Abstract

**Highlights:**

**What are the main findings?**

- A fully automated LabVIEW-LDV platform enables in-situ, zero-load extraction of nonlinear Rayleigh wave parameters in real time, without the need for post-processing.
- Higher-order nonlinear metrics (β_2_, β_3_) successfully detect early-stage microstructural fatigue long before macroscopic cracks emerge or linear acoustic properties change.

**What are the implications of the main findings?**

- The automated architecture eliminates operator bias and transient stress artifacts, offering a highly reproducible, low-latency solution for continuous Structural Health Monitoring (SHM).
- The system establishes a robust, real-time data-streaming foundation that is directly compatible with future AI-driven predictive maintenance and digital twin frameworks.

**Abstract:**

This paper presents a fully automated platform for real-time monitoring of fatigue-induced microstructural changes in metallic materials, using Rayleigh surface waves and Laser Doppler Vibrometry (LDV). The system integrates ultrasonic excitation, non-contact optical sensing, and high-speed signal processing in a unified LabVIEW environment. Rayleigh waves are generated via a contact transducer, while LDV captures surface vibrations with sub-nanometric velocity resolution, ensuring repeatability and eliminating coupling variability. The software automates synchronization, deterministic data acquisition, filtering, FFT analysis, and extraction of nonlinear coefficients (β_2_, β_3_) at high execution rates without the need for post-processing. Experimental validation under cyclic loading revealed a clear sensitivity hierarchy: the Rayleigh wave velocity remained invariant, the acoustic attenuation responded gradually, while the nonlinear parameters exhibited the earliest and steepest response to fatigue damage, confirming their superiority as early-stage indicators. The system offers low-latency timing, long-term stability, and modular design, establishing a robust data-streaming foundation that can support future integration with digital twin frameworks and machine learning models. Furthermore, the acoustic findings were successfully cross-validated using Infrared Thermography, which confirmed the critical damage transition phase. This work bridges nonlinear acoustics and software automation, providing a scalable diagnostic solution for predictive maintenance within structural health monitoring systems.

## 1. Introduction

Fatigue damage remains one of the most pervasive degradation mechanisms in metallic and composite structures subjected to cyclic loading, particularly in high-performance domains such as aerospace, energy, transportation, and defense systems [1,2,3]. It originates from microstructural changes—such as dislocation movement, persistent slip bands, microvoid coalescence, and microcrack formation—which evolve long before macroscale defects emerge [4,5,6]. Therefore, early detection of fatigue-induced degradation is critical for predictive maintenance, increased structural integrity, and avoidance of catastrophic failure [7,8].

Traditional nondestructive evaluation (NDE) techniques, including X-ray imaging, eddy current testing, acoustic emission, and linear ultrasonic testing, are typically effective only when damage has progressed to a detectable scale [9,10,11]. These linear techniques are sensitive to abrupt geometric discontinuities but fail to detect distributed or early-stage microstructural anomalies. In response to this limitation, nonlinear ultrasonics (NLU) has gained prominence as a promising approach capable of probing microstructural nonlinearities by leveraging higher-order harmonic generation and wave–defect interactions [12,13,14].

When ultrasonic waves propagate through a damaged medium, nonlinear scattering from fatigue-induced features—such as microcracks, grain boundary decohesion, and precipitate interfaces—produces measurable second- and third-order harmonic components [15,16,17]. These nonlinear parameters, commonly quantified using β_2_ and β_3_ coefficients, demonstrate strong correlations with accumulated fatigue damage and can serve as early-stage damage indicators [18,19,20].

Among the various wave modes employed in NLU, Rayleigh surface waves exhibit distinct advantages due to their confinement near the material surface and sensitivity to subsurface degradation [21,22]. First theoretically described by Lord Rayleigh in 1885 and later explored experimentally by Viktorov [23], these waves exhibit elliptical particle motion and decay exponentially with depth, making them ideal for detecting surface-near fatigue phenomena. Recent studies confirm that Rayleigh wave nonlinearity metrics can effectively monitor early plasticity evolution and microcrack formation in metallic specimens [24,25,26,27].

In terms of sensing, Laser Doppler Vibrometry (LDV) has emerged as a high-resolution, non-contact alternative to traditional piezoelectric receivers [28,29]. LDV provides highly accurate point-wise surface velocity data, is immune to couplant inconsistencies, and eliminates the need for mechanical contact, allowing better repeatability and minimal perturbation of the measured field [30,31,32]. Moreover, LDV systems are well suited for capturing higher harmonic content, especially when paired with advanced signal processing techniques [33,34].

However, despite advances in individual NLU and LDV technologies, current experimental implementations often require fragmented toolchains—comprising separate signal generation, data acquisition, post-processing, and user supervision—which reduce efficiency and repeatability [35]. For example, Walker et al. [16] employed nonlinear Rayleigh waves for fatigue evaluation in A36 steel but required specimen removal from the load frame for each measurement; Kim and Matlack [36] developed an in-situ nonlinear Rayleigh wave technique for stainless steel 316L using contact piezoelectric transducers, but the system required manual test interruption, performed all spectral analysis offline, and only extracted the second-order parameter β_2_; and Boccardi et al. [37] conducted in-situ nonlinear measurements during fatigue using separate hardware components (function generator, oscilloscope, contact transducers) with offline MATLAB-based post-processing R2013a. Manual calibration, operator bias, coupling variability inherent to contact transducers, and software heterogeneity further complicate fatigue monitoring in industrial contexts. Therefore, recent research efforts are moving towards integrated, automated platforms that unify sensing, excitation, analysis, and decision-making into cohesive environments [38,39,40].

LabVIEW-based architectures, particularly when synchronized with National Instruments (NI) hardware, offer an opportunity to realize this unification. By leveraging graphical programming, deterministic execution, and multithreaded data flow, real-time acquisition and nonlinear coefficient extraction become feasible within a single, high-speed execution loop [41]. Such frameworks enable in-situ monitoring during fatigue testing or operational loading, allowing for continuous assessment of material integrity and instantaneous SHM diagnostics [42,43,44].

Moreover, the fusion of NLU systems with digital twin paradigms is becoming a transformative direction for smart infrastructure and predictive health management [31]. While full digital twin implementation requires complex multi-physics modeling, the critical prerequisite is a reliable, continuous stream of physical data. The proposed automated platform fulfills this role by providing high-fidelity, real-time harmonic extraction that can act as a direct data-feeder for future digital twin or AI-driven diagnostic models [39].

This study presents a fully integrated diagnostic platform for fatigue detection based on Rayleigh wave excitation, LDV surface velocity measurement, and real-time harmonic analysis—all controlled within a unified LabVIEW environment. Unlike fragmented toolchains, the proposed system performs signal generation, acquisition, conditioning, and second/third harmonic analysis in real time. A scanning LDV captures Rayleigh responses, while a contact transducer excites controlled surface waves. Custom FFT routines extract β_2_ and β_3_ in situ, without post-processing or external scripting. Automation reduces operator variability, enhances consistency, and allows deterministic real-time diagnostics.

Validation experiments were conducted on aluminum specimens under low-cycle fatigue (LCF) loading. The extracted nonlinear parameters showed clear monotonic trends with accumulated fatigue cycles, confirming their efficacy as early indicators. The system exhibited robust timing, low noise, and high spatial resolution—key features for integration into structural health monitoring frameworks, including AI-enhanced and digital twin-based environments [45,46].

In summary, this work contributes to the advancement of real-time, software-defined nonlinear diagnostics for early fatigue detection. By combining theoretical insights, state-of-the-art sensing, and software unification, it lays a practical foundation for scalable SHM systems in complex industrial environments.

## 2. Materials and Methods

### 2.1. Rayleigh Wave Nonlinearity and Acoustic Metrics

The accumulation of microstructural damage during low-cycle fatigue (LCF) is characterized by alternating elastic and plastic deformations, leading to the formation of persistent slip bands (PSBs) and eventually microcracks. These microstructural anomalies alter the localized elastic properties of the material and interact nonlinearly with propagating acoustic waves. Rayleigh surface waves, due to their energy confinement within approximately one wavelength of the material’s surface, exhibit high sensitivity to the early stages of surface and subsurface degradation.

Unlike wave velocity, which depends on macroscopic elastic stiffness and remains insensitive to micro-scale damage, nonlinear acoustic parameters arise from the interaction between the propagating wave and sub-wavelength material discontinuities. Fatigue-induced microcracks exhibit asymmetric contact behaviour under acoustic loading—partially closing under compressive half-cycles and opening under tensile half-cycles—generating higher harmonics that are absent in intact material. Acoustic attenuation occupies an intermediate position: it responds to distributed scattering from diffuse microplasticity but with lower sensitivity and later onset than the nonlinear coefficients. The relative nonlinear parameters β_2_ and β_3_, defined as the ratios A_2_/A_1_^2^ and A_3_/A_1_^3^ respectively, quantify this harmonic generation and are therefore the earliest available indicators of progressive fatigue damage. When a monochromatic Rayleigh wave of fundamental frequency f propagates through a fatigue-damaged medium, the wave interacts with microstructural interfaces (e.g., crack opening and closing), distorting the waveform. This Contact Acoustic Nonlinearity (CAN) leads to the generation of higher-order harmonics. The acoustic nonlinearity parameters, primarily denoted as β_2_ (second-order) and β_3_ (third-order), serve as quantitative indicators of this degradation and are defined as:

(1) β2=A2A12

(2) β3=A3A13

where A_1_, A_2_, and A_3_ are the amplitudes of the fundamental, second, and third harmonic frequency components, respectively.

It should be noted that at the frequency-thickness product employed in this study, the propagating wave field may not be a purely surface-confined Rayleigh mode and could contain contributions from fundamental Lamb modes. However, two factors ensure that this does not affect the validity of the reported results. The wedge angle (65°), optimized via Snell’s law for the Rayleigh phase velocity of the specific alloy, provides preferential excitation of the Rayleigh mode. More importantly, because all nonlinear parameters are normalized against their pristine-state baseline values (β_2_/β_2(0)_, β_3_/β_3(0)_) under identical geometric and excitation conditions, any modal composition effects cancel systematically, and the measured parameter trends exclusively reflect the progressive microstructural degradation.

### 2.2. Specimen Preparation and Experimental Setup

**Figure 1:**
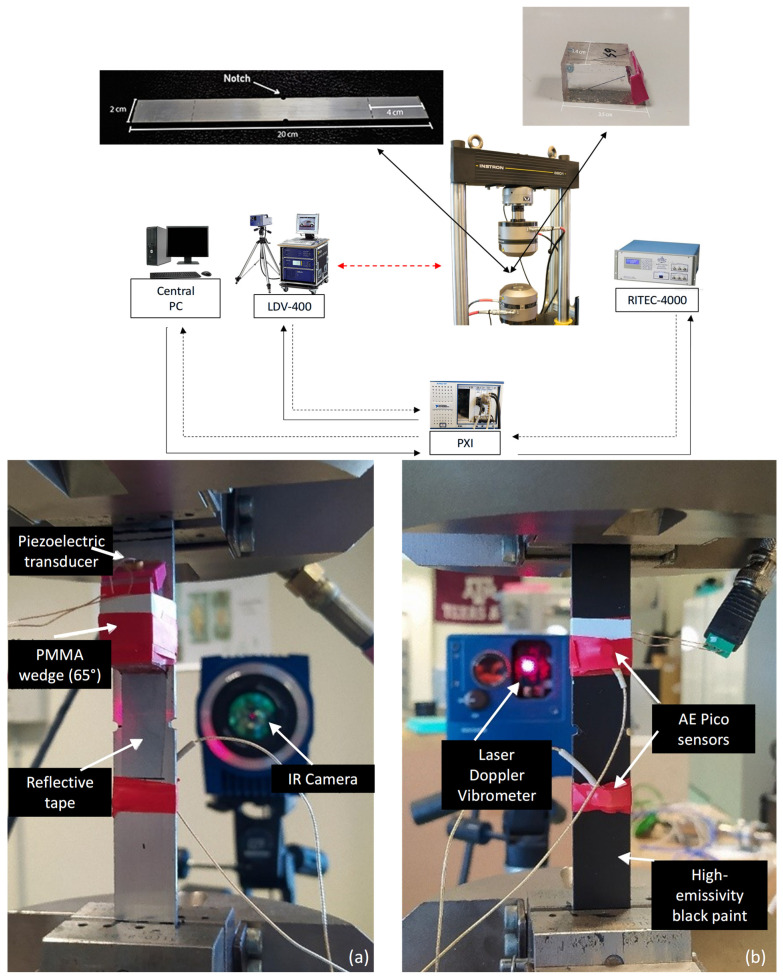
(**a**) Schematic representation of the automated experimental setup. (**b**) Close-up of the instrumented specimen mounted on the testing machine, showing the ultrasonic excitation wedge, LDV target area, and supplementary NDT sensors.

Experimental validation was conducted on Aluminum 6061-T6 specimens machined into a double-edge-notched (DEN) flat geometry with overall dimensions of 200 mm (length) × 20 mm (width) × 1.57 mm (thickness). To localize the stress concentration and dictate the area of fatigue damage evolution, two symmetric edge notches of 1 mm depth were machined at the center of the gauge section, producing a well-defined stress concentration that localized fatigue damage to a reproducible region. The complete experimental configuration is shown in Figure 1.

Surface acoustic waves were generated using a narrowband piezoelectric transducer (Boston Piezo-Optics Inc., Bellingham, MA, USA) with a central frequency of 1 MHz and a 0.25 mm element diameter. The transducer was permanently bonded to a custom Polymethyl Methacrylate (PMMA) wedge. Based on Snell’s law and preliminary pulse-echo velocity calibrations, the wedge angle was optimized at 65° to maximize mode conversion efficiency into Rayleigh waves for the specific aluminum alloy.

The out-of-plane surface velocity was recorded using a Polytec PSV-400 Laser Doppler Vibrometer (LDV) (Polytec GmbH, Waldbronn, Germany) equipped with a high-frequency displacement decoder. To cross-validate the macro-crack initiation phase, Infrared (IR) Thermography (Teledyne FLIR, Wilsonville, OR, USA) was deployed as an independent diagnostic tool. Acoustic Emission (AE) sensors (MISTRAS Group, Inc., Princeton, NJ, USA) were also attached but their results are not reported in this study.

### 2.3. Fatigue Loading Protocol

**Figure 2:**
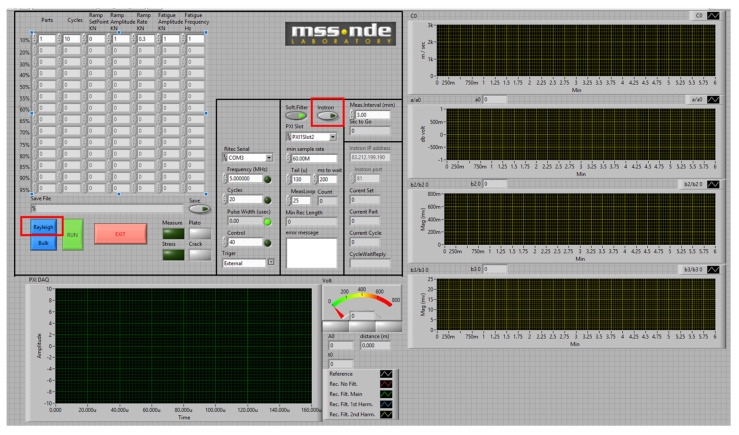
Graphical User Interfaces (GUIs) of the LabVIEW platform: main dashboard for real-time acoustic monitoring.

**Figure 3:**
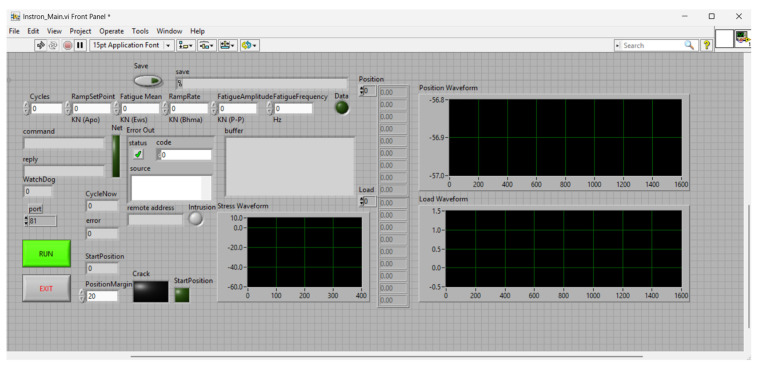
Graphical User Interfaces (GUIs) of the LabVIEW platform: integrated control panel for the Instron fatigue protocol.

Fatigue testing was performed using an Instron 8801 servo-hydraulic testing machine (Instron, Norwood, MA, USA). The specimens were subjected to tension-tension LCF conditions at a constant frequency of 5 Hz and a stress ratio of R = 0.1. The material’s Ultimate Tensile Strength (σ_UTS_) was determined as 346 MPa. To continuously monitor damage progression, a stepped loading protocol was implemented. The initial loading amplitude was set to 20% of the material’s Ultimate Tensile Strength (σ_UTS_) (i.e., 69 MPa). The load was systematically increased in increments of 10% σ_UTS_ (up to 173 MPa, i.e., 50% σ_UTS_) and subsequently by increments of ~17 MPa (5% σ_UTS_) until specimen fracture. Each stress block consisted of 6000 cycles. The main monitoring dashboard and the fatigue control panel are presented in Figure 2 and Figure 3, respectively.

### 2.4. Integrated Hardware Architecture and Process Synchronization

To ensure deterministic execution and eliminate operator-induced variations, the entire experimental procedure was orchestrated by a custom primary-secondary architecture developed in LabVIEW 2014 (National Instruments Corp., Austin, TX, USA). The hardware backbone consisted of a National Instruments PXI-1071 (National Instruments Corp., Austin, TX, USA) chassis, which integrated a controller (PXIe-8360 (National Instruments Corp., Austin, TX, USA)), a serial interface (PXIe-8430/2) to govern the RITEC generator (RITEC, Inc., Warwick, RI, USA), and a high-speed digitizer (PXIe-5105) operating at a 60 MS/s sampling rate to capture the LDV responses with high temporal resolution.

Unlike standalone NDT setups, the proposed platform introduced a continuous, bidirectional TCP/IP communication link with the Instron 8801 servo-hydraulic controller. The software autonomously managed the low-cycle fatigue (LCF) protocol through a hierarchical looping structure. Upon the completion of each 6000-cycle block, the LabVIEW 2014 (National Instruments Corp., Austin, TX, USA) primary program commanded the testing machine to halt cycling and gradually ramp down to a confirmed zero-load state (0 kN).

This automated zero-load pause was a critical methodological step, ensuring that the subsequent acoustic measurements captured permanent fatigue-induced microstructural degradation, completely decoupled from transient stress–strain effects. Once the zero-load condition was confirmed via the TCP/IP feedback, the software synchronously triggered the RITEC pulser to excite the Rayleigh waves and armed the digitizer to record the out-of-plane velocity from the LDV. Following data acquisition, the system automatically instructed the Instron machine to resume the next cyclic loading block.

Crucially, to establish the baseline acoustic parameters, the pristine specimen was initially scanned at two distinct spatial points: exactly beneath the wedge (excitation point) and at a remote point within the highly stressed gauge section. For all subsequent zero-load measurements between fatigue blocks, the LDV beam remained focused solely on the remote gauge point. This approach eliminated repositioning errors and allowed the relative variations in the parameters to be tracked dynamically against the initial dual-point baseline.

This integrated architecture represents a methodological departure from all existing nonlinear ultrasonic fatigue implementations reported in the literature. Current state-of-the-art approaches rely on fragmented toolchains with one or more of the following limitations: contact piezoelectric reception susceptible to coupling variability [16,19,36,37], manual test interruption for acoustic measurements [36,37], measurements performed under applied stress rather than at a controlled zero-load state [36], offline post-processing in external software environments [16,36,37,47], and extraction limited to the second-order parameter β_2_ alone [16,19,36]. The proposed platform addresses all of these limitations simultaneously through three key innovations: (i) non-contact LDV reception, which eliminates coupling variability and repositioning errors that directly contaminate harmonic amplitude measurements—a concern highlighted as critical by Matlack et al. [48]; (ii) automated zero-load extraction via bidirectional TCP/IP synchronization with the servo-hydraulic machine, ensuring that all measured harmonic content reflects permanent microstructural degradation decoupled from transient stress-induced nonlinearity; and (iii) real-time FFT-based harmonic computation within a single LabVIEW execution loop, eliminating post-processing latency. To the best of the authors’ knowledge, no existing study combines non-contact detection, automated zero-load synchronization, and real-time dual-parameter (β_2_ and β_3_) extraction within a unified software environment.

### 2.5. Real-Time Signal Processing and Nonlinear Feature Extraction

Immediately following waveform acquisition, the software executed a rigorous, real-time signal processing pipeline to extract both linear and nonlinear acoustic indicators, bypassing the need for offline analysis.

The processing sequence consisted of the following stages:

- **Digital Conditioning:** The raw optical signal was subjected to a digital Butterworth band-pass filter implemented within the LabVIEW environment. The Butterworth topology was specifically selected for its maximally flat amplitude response in the passband, thereby preventing artificial amplitude distortions that could compromise the higher-order harmonic calculations. The filter provides operator-selectable order and an adjustable passband whose center frequency and half-bandwidth are controlled dynamically via dedicated interface controls: the half-bandwidth parameter defines the symmetric passband extent (in Hz) around the excitation frequency, while the order parameter governs the rolloff steepness—lower values yield smoother transitions with greater temporal stability, whereas higher values offer sharper spectral selectivity at the expense of transient ringing. During all fatigue experiments reported herein, the filter configuration was fixed at system commissioning and held strictly constant across every acquisition stage, thereby eliminating inter-stage processing variability and ensuring that spectral differences between measurements arise solely from material-state evolution.
- **Temporal Windowing and Energy Validation:** To isolate the primary Rayleigh wave packet from potential edge reflections or mode-converted signals, a dynamic gating algorithm was applied using a 33% peak-amplitude threshold. This threshold was selected on the basis of the system’s signal-to-noise characteristics: for the metallic specimens and LDV configuration employed in this study, the baseline noise floor typically resides below 10% of the peak signal amplitude. A threshold at 33% therefore provides a detection margin of approximately 3:1 above the noise floor, ensuring reliable wave-packet onset identification while excluding pre-arrival noise and trailing-edge reflections. The suitability of this material-specific threshold has been further confirmed across the authors’ broader experimental program, which includes cementitious composites requiring a fundamentally different dual-threshold strategy (10% + 50%) due to their higher attenuation and heterogeneity [33]. The consistent application of the 33% threshold across all metallic specimens ensures that the gating window definition does not introduce systematic bias between fatigue stages.

To mathematically validate the integrity of the gated segment, an energy-based refinement was implemented utilizing Parseval’s theorem, which establishes the fundamental energy equivalence between the time and frequency domains:

(3) $$\sum_{n=0}^{N-1}{\left|x\left[n\right]\right|}^{2}=\frac{1}{N}\sum_{k=0}^{N-1}{\left|X\left[k\right]\right|}^{2},$$

where x[n] represents the discrete time-domain velocity signal, X[k] denotes its DFT, and N is the total number of acquired samples. Based on this energy conservation principle, the cumulative energy curve of the waveform was computed and the processing window was strictly truncated at the 50% cumulative-energy point, with boundaries further refined by a ±20% sample margin to accommodate residual ringing. This two-stage approach—amplitude-based gating followed by energy-based validation—ensures that the retained waveform segment contains the dominant acoustic information while systematically excluding low-energy trailing oscillations that could contaminate the harmonic extraction. All processing parameters were determined during system commissioning and remained constant throughout the experimental campaign.

**Figure 4:**
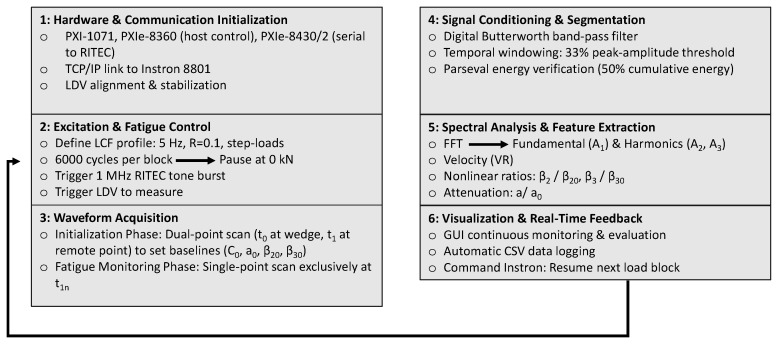
Schematic of the software workflow—from initialization and excitation control through acquisition, conditioning, spectral analysis, velocity and attenuation estimation, nonlinear metrics, and visualization—with a real-time feedback loop enabling fully automated, operator-independent nonlinear acoustic monitoring.

- **Harmonic Evaluation:** The energy-validated time window was transformed using a Fast Fourier Transform (FFT). An automated peak-tracking routine identified the amplitudes of the fundamental (A_1_), second (A_2_), and third (A_3_) harmonics. The nonlinear acoustic parameters (β_2_ and β_3_) were then computed. To track degradation over the specimen’s fatigue life, these parameters were normalized against their initial baseline values (β_2(0)_, β_3(0)_) recorded at the pristine state.
- **Velocity and Attenuation Metrics:** The Rayleigh wave velocity (V_R_) was calculated based on the time-of-flight (Δt). To ensure robust leading-edge detection regardless of signal attenuation, Δt was determined by applying a strict 33% amplitude threshold to the wave packets. Concurrently, the material’s acoustic attenuation (α) was dynamically quantified by calculating the input-to-output amplitude drop. The attenuation in decibels (dB) was computed as:
  (4) $$\alpha =-20\log_{10}\left(V_{out}/V_{in}\right),$$
  where V_in_ and V_out_ represent the peak amplitudes of the excitation and the received signals, respectively. To track progressive material damping, this value was normalized against the baseline attenuation (α_0_) recorded at the pristine state. The complete signal processing pipeline is illustrated schematically in Figure 4.

In summary, the platform receives as inputs the operator-defined excitation parameters (frequency, amplitude, number of cycles), the LDV-acquired surface velocity waveform, and the fatigue loading state communicated via TCP/IP from the servo-hydraulic controller. It processes these inputs through the automated pipeline described above and produces, in real time, the following diagnostic outputs: the Rayleigh wave velocity (VR), the acoustic attenuation (α/α_0_), the nonlinear parameters (β_2_/β_2(0)_, β_3_/β_3(0)_), and archived time-stamped records for each fatigue stage. This closed-loop, single-environment architecture ensures a continuous, operator-independent data stream suitable for integration with predictive maintenance and digital twin frameworks.

## 3. Experimental Validation

### 3.1. System Stability and Linearity Assessment

Prior to monitoring fatigue evolution, the intrinsic stability and linearity of the developed hardware-software architecture were rigorously evaluated. It is imperative in nonlinear ultrasonics to ensure that the measured harmonic generation originates exclusively from the material’s microstructural state rather than from instrumentation artifacts or electronic distortion.

To validate long-term stability, continuous baseline measurements were conducted over a 7-h period on an un-fatigued 14.8 mm thick steel reference specimen under room temperature conditions. The automated LDV platform recorded the surface velocity and extracted the spectral components continuously.

**Figure 5:**
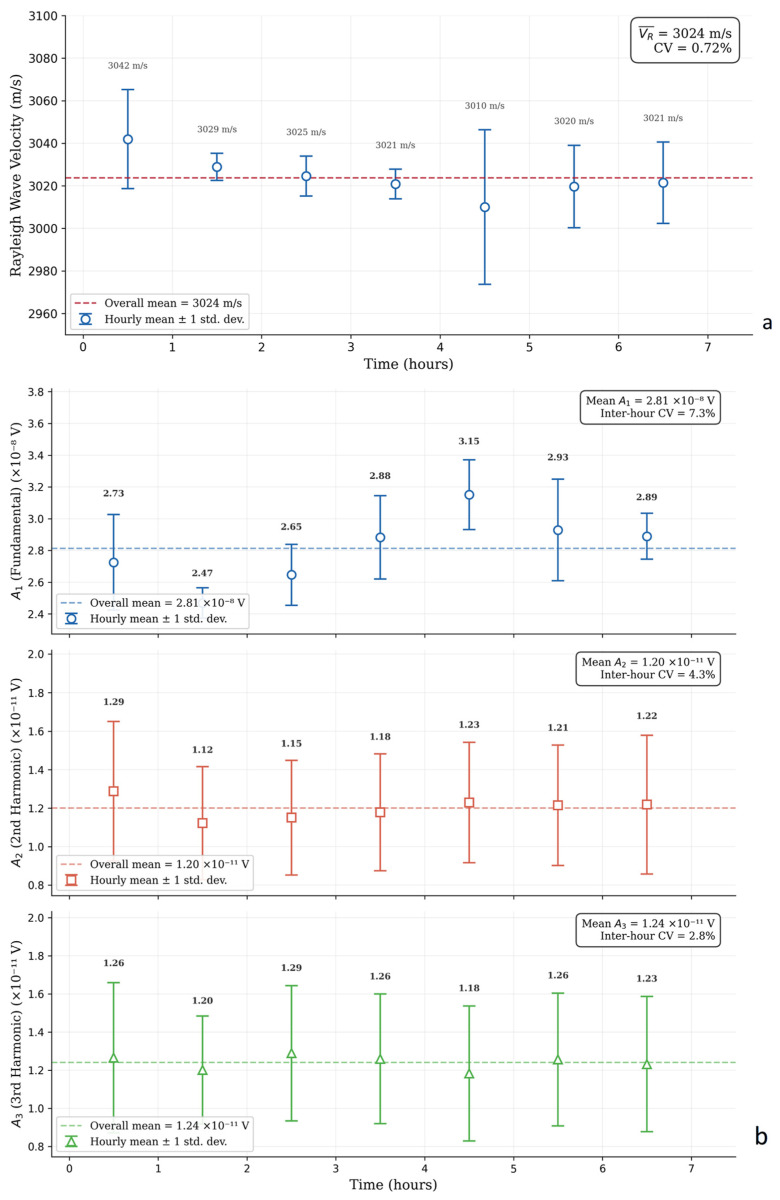
Long-term stability test of the measurement system at room temperature: (**a**) Rayleigh wave velocity monitoring, (**b**) Amplitudes of the fundamental, second, and third harmonics.

The data were segmented into hourly intervals and evaluated statistically (Figure 5). The Rayleigh wave velocity remained stable throughout the 7-h acquisition, with a mean of 3024 m/s and a coefficient of variation (CV) of 0.72%. The harmonic amplitudes A_1_, A_2_, and A_3_ exhibited inter-hour CVs of 7.3%, 4.3%, and 2.8%, respectively, confirming the absence of systematic drift in the measurement chain.

**Figure 6:**
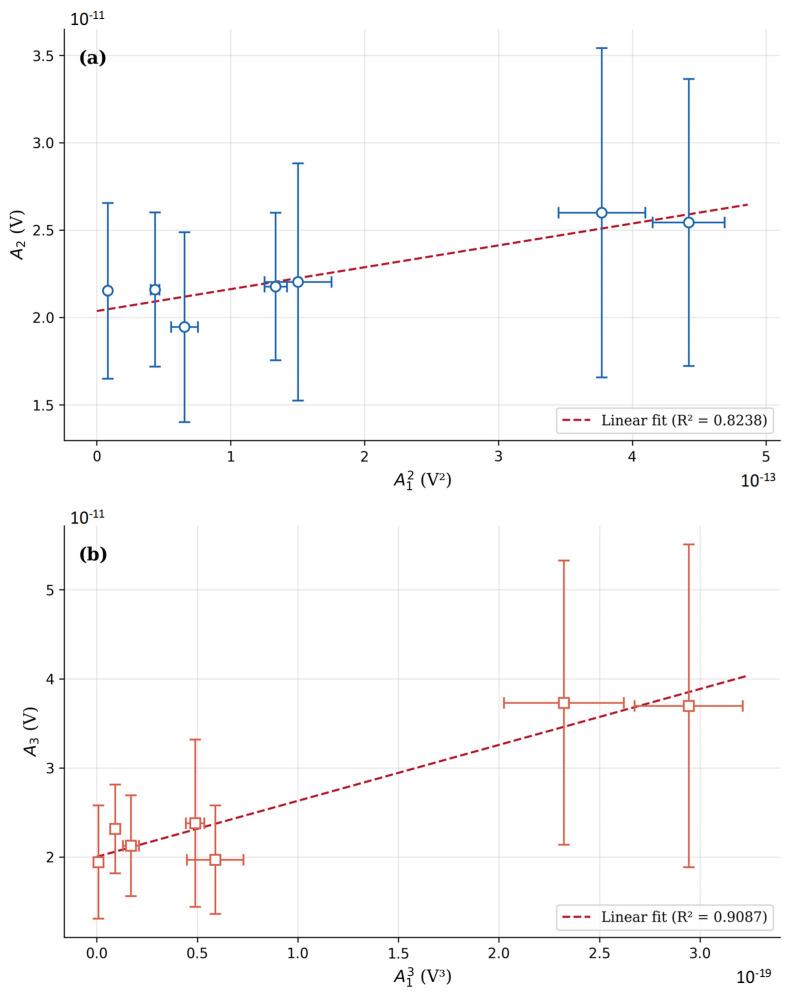
Linearity analysis of the measurement system: (**a**) A_2_ versus A_1_^2^ for parameter β_2_; (**b**) A_3_ versus A_1_^3^ for parameter β_3_.

Furthermore, to verify the linearity of the measurement chain, the amplitude of the fundamental excitation was incrementally varied. According to classical nonlinear acoustic theory, for a purely linear system, the normalized ratios A_2_/A_1_^2^ and A_3_/A_1_^3^ should remain constant regardless of the excitation voltage. Linear regression analysis of the measured data yielded coefficients of determination of R^2^ = 0.8237 for the second-order and R^2^ = 0.9087 for the third-order ratio. While in purely electrical calibrations higher R^2^ values are typical, the present non-contact optical configuration measures higher harmonics whose amplitudes in the pristine material state are vanishingly small, residing near the LDV’s optical sensitivity limits. The ultrasonic excitation, generated by a single-crystal piezoelectric element with inherently low harmonic distortion, ensures that the dominant harmonic content originates from wave–material interaction rather than from the excitation electronics. The moderate deviation from ideal linearity is characterized by random scatter rather than systematic nonlinear trends, as confirmed by the absence of systematic drift during the 7-h continuous stability assessment (Figure 5). Crucially, the magnitude of harmonic generation observed during fatigue testing (Section 3.2) exceeds the baseline fluctuations by multiples of the pristine-state values, confirming that the measured escalations in β_2_ and β_3_ are driven by material-state changes—principally crack-face contact nonlinearity—and not by equipment artifacts. The linearity analysis results are presented in Figure 6.

### 3.2. Real-Time Nonlinear Acoustic Evaluation of Metallic Materials

**Figure 7:**
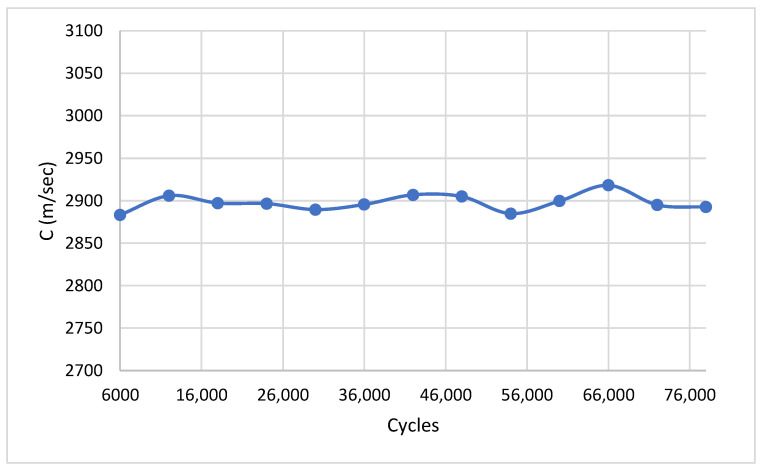
Rayleigh wave velocity versus fatigue cycles for Aluminum 6061-T6.

**Figure 8:**
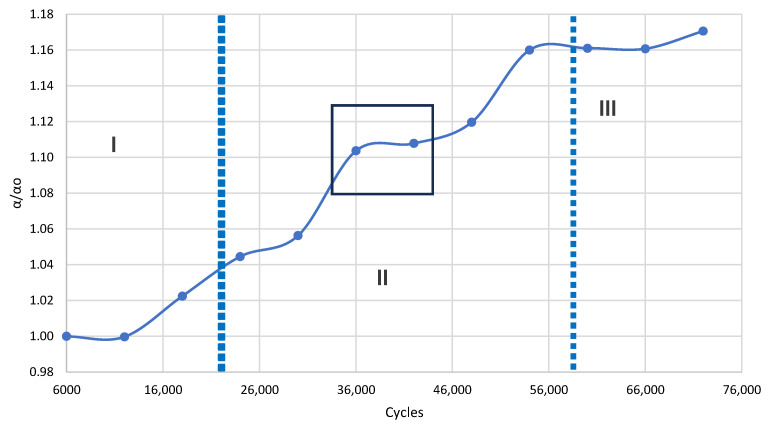
Normalized acoustic attenuation as a function of fatigue cycles for Aluminum 6061-T6.

**Figure 9:**
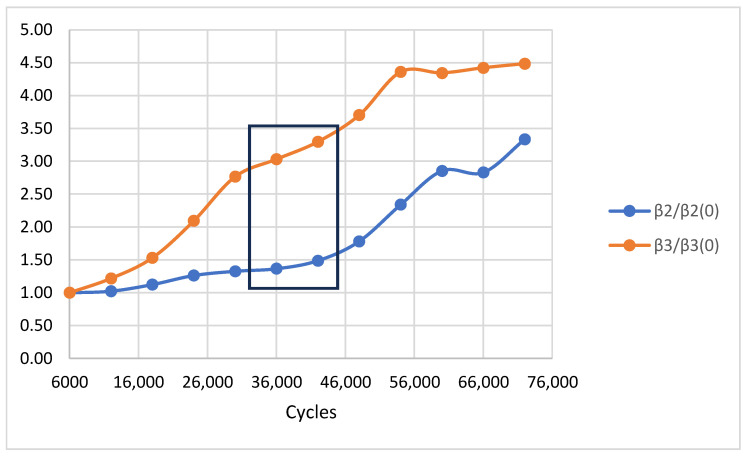
Normalized nonlinear parameters versus fatigue cycles for Aluminum 6061-T6.

The fully automated platform was deployed to monitor the low-cycle fatigue progression in Aluminum 6061-T6 specimens. An adequate number of specimens was tested to verify the repeatability of the observed trends; however, a detailed statistical treatment of fatigue life scatter (mean values, standard deviations) is intentionally outside the scope of this work, whose primary contribution is the demonstration and validation of the automated diagnostic platform. Data acquisition was performed at 6000-cycle intervals exclusively at the 0 kN zero-load state. The evolution of the Rayleigh wave velocity throughout the fatigue life is shown in Figure 7. The normalized acoustic attenuation is presented in Figure 8, and the normalized nonlinear parameters are shown in Figure 9.

The experimental results reveal a clear sensitivity hierarchy among the three measured acoustic parameters—velocity, attenuation, and nonlinear coefficients—which is detailed in the following paragraphs.

Throughout the entire fatigue lifespan, the absolute Rayleigh wave velocity (V_R_) remained remarkably stable at approximately 2920 m/s (fluctuations < ±1%), exhibiting excellent agreement with the theoretical velocity calculated from the material’s elastic constants. This stability indicates that the macroscopic elastic properties defining the wave speed are not significantly altered by localized microstructural damage until catastrophic failure is imminent.

In contrast, the normalized acoustic attenuation (α/α_0_) exhibited a characteristic three-stage sigmoidal evolution. During the initial stage (up to ~20,000 cycles), the attenuation remained constant, reflecting the purely elastic response of the pristine microstructure. The second, transitional phase (20,000–58,000 cycles) was marked by a gradual increase in attenuation, correlating with the onset of localized plastic deformation and microcrack nucleation, which act as scattering centers. Finally, beyond 60,000 cycles, the attenuation gradient steepened sharply, culminating in a 10–20% increase prior to failure.

The most sensitive indicators of damage were the normalized nonlinear parameters (β_2_/β_2(0)_ and β_3_/β_3(0)_). Both indices exhibited a monotonic, three-stage increase that preceded macroscopic degradation. During the initial 20,000 cycles, the parameters hovered near unity, indicating reversible micro-plasticity without persistent crack formation. In the intermediate stage (20,000–50,000 cycles), the generation of higher-order harmonics surged. Notably, the third-order parameter (β_3_/β_30_) exhibited a much steeper and earlier escalation compared to the second-order parameter. This amplified sensitivity is attributed to the “crack breathing” phenomenon, where the cubic nonlinearity responds more aggressively to the asymmetric contact mechanics of developing microcracks. In the final stage (>50,000 cycles), the nonlinear parameters approached a plateau, likely indicating saturation of crack density within the ultrasonic interaction volume just before ultimate fracture; this interpretation warrants further corroboration via post-mortem microscopy.

### 3.3. Multi-Modal Cross-Validation via Infrared Thermography

To independently corroborate the critical damage thresholds identified by the nonlinear acoustic platform, Infrared (IR) Thermography was employed concurrently to monitor the dissipated thermal energy during the cyclic loading.

**Figure 10:**
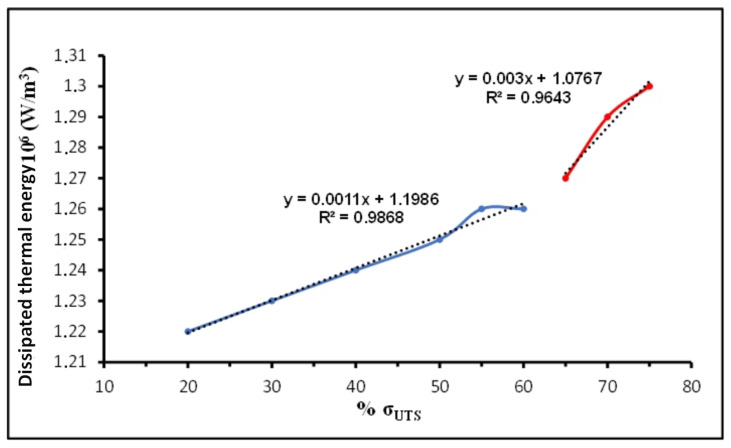
Evolution of the dissipated thermal energy monitored via IR Thermography.

As illustrated in Figure 10, the thermographic profile showed a distinct, rapid change in thermal response over the range of 36,000 to 42,000 fatigue cycles (~65% σ_UTS_). In this transition window, the dissipated energy values showed a measurable increase, but the most significant indicator was the change in the dissipation rate (slope). Linear regression analysis reveals that the slope coefficient increased from 0.0011 (R^2^ = 0.9868) during the early fatigue life to 0.003 (R^2^ = 0.9643) after the critical threshold. This nearly threefold increase in the dissipation rate is indicative of increased internal friction arising from localized plasticity and active microcrack nucleation.

This thermal transition—indicative of increased internal friction due to localized plasticity and active microcrack nucleation—closely coincides in time with the pronounced escalation observed in both the acoustic attenuation and the nonlinear acoustic parameters (β_2_, β_3_). The temporal alignment of the dissipated thermal energy surge with the acoustic nonlinearity spike provides robust multi-modal validation. It confirms that the automated LDV-LabVIEW platform accurately captures the transition from reversible microstructural fatigue to irreversible macro-scale damage.

## 4. Discussion

The three-stage sigmoidal behavior of β_2_ and β_3_ aligns well with the established physical models of fatigue: reversible micro-plasticity, irreversible microcrack accumulation, and final macro-crack propagation. The independent validation using Infrared Thermography provided strong corroboration of the critical damage transition identified by the nonlinear acoustic parameters, confirming that the onset of significant thermal dissipation coincides temporally with the steepest gradient region of both β_2_ and β_3_.

The experimental results collectively validate the effectiveness of the automated LabVIEW–LDV architecture as a real-time fatigue diagnostic tool. The most instructive finding is the stark contrast in sensitivity between the linear and nonlinear parameters. The Rayleigh wave velocity remained essentially invariant at approximately 2920 m/s throughout the entire fatigue life, consistent with the expectation that macroscopic elastic moduli are insensitive to early-stage microstructural changes such as dislocation rearrangement and nascent slip band formation. This observation is in agreement with results reported by Walker et al. [16] for A36 steel and by Pfeifer et al. [19] for X52 pipeline material, where Rayleigh wave velocity similarly showed negligible variation prior to macro-crack formation, reinforcing the conclusion that velocity-based linear metrics are insufficient as standalone early-damage indicators.

In contrast, the normalized nonlinear parameters β_2_/β_2(0)_ and β_3_/β_3(0)_ exhibited a pronounced three-stage evolution physically consistent with established low-cycle fatigue mechanics. The initial stage of near-unity values reflects reversible micro-plasticity in which dislocation structures have not yet stabilized into persistent slip bands. The steep intermediate escalation (20,000–50,000 cycles) corresponds to irreversible microcrack nucleation, where crack-face interactions generate measurable harmonic distortion. This sensitivity is consistent with findings reported by Kim et al. [11] for nickel-base superalloys and by Park et al. [15] for aluminum under bending fatigue, both of which showed nonlinear parameters escalating well before any linear indicator responded. The eventual plateau beyond 50,000 cycles is interpreted as saturation of the crack density within the acoustic interaction volume, a behavior also described by Ding et al. [20] in the context of Lamb wave nonlinearity in cyclically loaded metallic specimens.

The superior early-stage sensitivity of the third-order parameter β_3_ relative to β_2_ merits further physical discussion. In classical nonlinear acoustic theory, β_2_ is governed by second-order elastic nonlinearity arising from lattice anharmonicity and dislocation density, while β_3_ is amplified by third-order elastic constants and is particularly sensitive to asymmetric crack-face contact mechanics—the so-called ‘crack breathing’ mechanism. In the present double-edge-notched geometry, microcrack faces at the notch root experience asymmetric displacement fields even at zero mean load due to local residual stress concentrations, rendering them highly effective generators of odd-order harmonics. This interpretation is consistent with the analysis by Lissenden [21], who identified third-order nonlinearity as the dominant indicator in specimens with localized crack populations. The earlier and steeper escalation of β_3_ therefore represents a genuine physical advantage for notched specimen geometries and suggests that third-order analysis should be prioritized in future fatigue monitoring protocols.

The multi-modal cross-validation with Infrared Thermography provides strong independent corroboration of the critical damage transition identified acoustically. The thermographic inflection at 36,000–42,000 cycles, corresponding to approximately 65% σ_UTS_, coincides with the steepest gradient region of both β_2_ and β_3_. The physical basis for this alignment is well established: the rapid increase in dissipated thermal energy reflects elevated internal friction from crack-face rubbing and accelerated dislocation motion—the same microstructural processes that drive harmonic generation. Notably, the attenuation parameter begins its transitional phase at a somewhat earlier cycle count (~20,000 cycles), consistent with its sensitivity to distributed scattering from diffuse microplasticity, while the nonlinear parameters respond most steeply at 20,000–50,000 cycles when discrete microcracks dominate. The temporal convergence of all three indicators at the 36,000–42,000 cycle window confirms that this range represents the critical transition from reversible to irreversible damage, and the multi-modal agreement rules out instrumentation artefacts as an alternative explanation.

From an automation and systems perspective, the deterministic zero-load measurement protocol introduced in this work represents a meaningful methodological advancement over all existing in-situ NLU implementations. Kim and Matlack [36] performed their in-situ nonlinear Rayleigh wave measurements under applied stress, meaning that the measured β values contained both permanent microstructural and transient stress-dependent contributions—a confounding factor that the present zero-load protocol eliminates. Walker et al. [16] avoided this issue by removing specimens from the load frame entirely, but at the cost of precluding continuous monitoring. Boccardi et al. [37] performed in-situ measurements during fatigue but relied on contact piezoelectric transducers and offline post-processing, requiring manual intervention at each measurement stage. By commanding the servo-hydraulic machine to a confirmed 0 kN state prior to every acoustic acquisition via bidirectional TCP/IP synchronization, the present platform ensures that all measured harmonic content reflects permanent microstructural state changes rather than transient stress-induced nonlinearity. This is particularly important for the accurate extraction of β_3_, which is more susceptible to stress-dependent variations than β_2_ [21]. The real-time FFT-based harmonic extraction eliminates the post-processing latency inherent in fragmented toolchains and enables continuous, in-situ diagnostic assessment without operator intervention. This capability directly addresses the integration gap identified in the literature between laboratory-grade NLU precision and the deterministic, low-latency requirements of industrial SHM.

Several limitations of the present study should be acknowledged to guide future work. The experimental validation was conducted on a single alloy system (Aluminum 6061-T6) under tension-tension LCF at a fixed stress ratio R = 0.1; the generalizability of the three-stage parameter evolution to other alloys, multiaxial loading, or variable-amplitude spectra has not been established here. The system stability assessment was performed on a steel reference specimen rather than on the test alloy, and the Butterworth filter bandwidth and 33% amplitude gating threshold were set empirically without a formal sensitivity analysis. Furthermore, the primary focus of this work is the demonstration of the automated platform rather than a full statistical fatigue characterization; a multi-specimen statistical campaign quantifying parameter variability is planned as follow-on work. Future developments will also focus on integrating the real-time data stream with machine learning models for automated damage-state classification and remaining useful life estimation.

## 5. Conclusions

This study introduced a fully automated, real-time diagnostic platform for the continuous monitoring of fatigue degradation in metallic materials. By integrating Rayleigh surface wave excitation, non-contact Laser Doppler Vibrometry, and a deterministic TCP/IP handshake with a servo-hydraulic testing machine, the custom LabVIEW architecture enabled autonomous, operator-independent nonlinear acoustic evaluations throughout the entire fatigue lifespan of the specimen.

The platform’s methodological integrity was first established through rigorous baseline characterisation. Long-term stability measurements confirmed the repeatability of the LDV-based acquisition chain, while linearity analysis across a range of excitation amplitudes verified that the extracted harmonic parameters reflect authentic material state rather than instrumentation artefacts—a prerequisite for meaningful nonlinear acoustic diagnostics.

The experimental results demonstrated a clear hierarchy of damage sensitivity among the measured parameters. The absolute Rayleigh wave velocity remained essentially invariant throughout fatigue life, confirming that macroscopic elastic properties are insensitive to early microstructural degradation. In contrast, the normalised nonlinear parameters β_2_ and β_3_ exhibited a pronounced, monotonic three-stage evolution—from a stable baseline during reversible micro-plasticity, through a steep escalation driven by microcrack nucleation and growth, to a final plateau associated with crack density saturation prior to fracture. The third-order parameter proved particularly discriminating, responding earlier and more steeply to developing damage than its second-order counterpart, consistent with the higher sensitivity of cubic nonlinearity to asymmetric crack-face contact mechanics. This sensitivity hierarchy underscores the necessity of higher-harmonic analysis for early-stage fatigue detection where linear velocity measurements remain insensitive.

Independent corroboration via Infrared Thermography strengthened the diagnostic conclusions. The thermographic transition—marking the onset of significant internal heat dissipation due to localised plasticity and active microcrack formation—aligned closely with the steep gradient region in both the acoustic attenuation and the nonlinear parameters, confirming that the automated platform accurately resolves the critical transition from reversible microstructural fatigue to irreversible macro-scale damage at approximately 65% σ_UTS_.

From a systems perspective, the automated zero-load measurement protocol was central to the platform’s reliability. By decoupling acoustic measurements from transient stress–strain fields and eliminating manual repositioning, the architecture produced high-density, reproducible data streams that are directly compatible with data-driven analysis frameworks. The demonstrated integration of hardware orchestration, real-time signal processing, and continuous data archiving within a unified software environment establishes a practical foundation for next-generation structural health monitoring. Future work will focus on leveraging this high-fidelity data stream to train machine learning models and populate digital twin frameworks, with the ultimate aim of enabling predictive maintenance and remaining useful life estimation in critical engineering infrastructure.

## Data Availability

The raw data supporting the conclusions of this article will be made available by the authors on request.
